# Atherothrombosis in Acute Coronary Syndromes—From Mechanistic Insights to Targeted Therapies

**DOI:** 10.3390/cells10040865

**Published:** 2021-04-10

**Authors:** Chinmay Khandkar, Mahesh V. Madhavan, James C. Weaver, David S. Celermajer, Keyvan Karimi Galougahi

**Affiliations:** 1 Department of Cardiology, Orange Base Hospital, Orange, NSW 2800, Australia; ckha7179@uni.sydney.edu.au; 2 Faculty of Medicine and Health, University of Sydney, Sydney, NSW 2008, Australia; James.Weaver@health.nsw.gov.au (J.C.W.); David.Celermajer@health.nsw.gov.au (D.S.C.); 3 New York Presbyterian Hospital/Columbia University Irving Medical Center, New York, NY 10032, USA; mvm2122@cumc.columbia.edu; 4 Clinical Trials Center, Cardiovascular Research Foundation, New York, NY 10019, USA; 5 Department of Cardiology, Royal Prince Alfred Hospital, Sydney, NSW 2050, Australia; 6 Heart Research Institute, Sydney, NSW 2042, Australia

**Keywords:** acute coronary syndromes, optical coherence tomography, plaque rupture, plaque erosion, calcified nodule, percutaneous coronary intervention

## Abstract

The atherothrombotic substrates for acute coronary syndromes (ACS) consist of plaque ruptures, erosions and calcified nodules, while the non-atherothrombotic etiologies, such as spontaneous coronary artery dissection, coronary artery spasm and coronary embolism are the rarer causes of ACS. The purpose of this comprehensive review is to (1) summarize the histopathologic insights into the atherothrombotic plaque subtypes in acute ACS from postmortem studies; (2) provide a brief overview of atherogenesis, while mainly focusing on the events that lead to plaque destabilization and disruption; (3) summarize mechanistic data from clinical studies that have used intravascular imaging, including high-resolution optical coherence tomography, to assess culprit plaque morphology and its underlying pathobiology, especially the newly described role of innate and adaptive immunity in ACS secondary to plaque erosion; (4) discuss the utility of intravascular imaging for effective treatment of patients presenting with ACS by percutaneous coronary intervention; and (5) discuss the opportunities that these mechanistic and imaging insights may provide for more individualized treatment of patients with ACS.

## 1. Introduction

Major insights into the pathobiology of coronary atherosclerosis have been gained from postmortem histopathological studies [1,2,3]. Advances in intravascular imaging technology (e.g., intravascular ultrasound (IVUS) and more recently optical coherence tomography (OCT)) have allowed for in vivo high-resolution imaging and detailed analysis of the atherosclerotic plaque morphology. These imaging modalities, combined with mechanistic biomarker and clinical data, have led to a more in-depth understanding of the natural history of atherosclerosis, including identification of the major triggers and substrates for atherothrombotic events. In this review, we provide an overview on the pathobiology of the three major substrates of acute coronary syndromes (ACS)—plaque rupture, plaque erosion and calcified nodules—and discuss the potential therapeutic considerations that may stem from these mechanistic insights, particularly as they pertain to personalized treatment approaches.

## 2. Development and Progression of the Atherosclerotic Plaques

During the early development of atherosclerotic plaques, increased concentrations of apoprotein-β-containing lipoproteins, most commonly low-density lipoprotein, accumulate in the arterial intima via binding with intimal proteoglycans [4]. The lipoproteins oxidized within intima, or taken up from plasma in their oxidized form, are scavenged by macrophages, resulting in foam cell formation and thereby formation of the fatty streaks—the histopathologic hallmark of early atherosclerosis. Further infiltration of medial smooth muscle cells (SMCs) and T-helper 1 cells amplifies this response [5]. Some of these fatty streaks develop into progressive atherosclerotic lesions with continued accumulation of lipid-rich material in the intima. Foam cells then undergo apoptosis due to prolonged endoplasmic reticulum stress and are inadequately cleared, resulting in cellular necrosis [6,7]. This process, over time, results in the formation of a necrotic core [6]. A fibrous layer formed by intimal SMCs creates a fibrous cap, which separates the necrotic lipid-rich core from the intimal endothelium. Advanced plaques also exhibit neoangiogenesis, mainly originating from the adventitial vasa vasorum, which supply both nutrition and inflammatory cells to further evolve the plaques [4]. These neo-vessels are fragile due to compromised structural integrity and are highly susceptible to leakage and intraplaque hemorrhage, thus further recruiting inflammatory infiltrates [4]. These processes, in combination, lead to the development of enlarging intraplaque necrotic lipid pools, which ultimately occupy a major part of the arterial wall, leading initially to expansive or positive remodeling [8]. With further progression, however, the plaque begins to extend into the lumen, leading to progressive stenosis (i.e., negative remodeling) [8].

There is an increasing body of evidence demonstrating the role of inflammation in atherogenesis. The NOD-like receptor protein 3 (NLRP3) inflammasome is a cytosolic complex, present in leukocytes, which is a key mediator in atherogenesis. Damage associated molecular proteins, such as cholesterol crystals from apoptotic macrophages, prime the inflammasome complex, leading to the assembly of the NLRP3 complex and activation of the procaspase domain to caspase-1 [9,10]. Caspase-1 cleaves pro-interleukin-1 β (pro-IL-1β) into the effector molecule IL-1β [11]. This atherogenic cytokine has been shown to induce expression of endothelin-1 and other adhesion molecules to promote leukocyte migration into the atheromatous plaque and perpetuate the inflammatory response [12]. It has also been shown to enhance the lifespan of monocytes and neutrophils, as well as to increase generation of the lytic enzymes [13,14,15]. As pro-IL-1β is expressed at low-levels at baseline, initial induction is required prior to caspase-1 cleavage. Dying granulocytes release histones from DNA and myeloperoxidase (MPO), which form a mesh-like network with fibrin strands termed neutrophil extracellular traps (NETs) in a process known as NETosis [16,17]. NETs are released by neutrophils that are exposed to cholesterol crystals, which could subsequently colocalize to the nearby inflammatory macrophages to prime IL-1β expression [18].

## 3. Plaque Destabilization and Disruption

Activated macrophages and T-helper 1 cells infiltrate the collagen cap, thereby producing metalloproteinases (MMPs) and several inflammatory cytokines (e.g., interferon-γ (IFN-γ) and tumor necrosis factor (TNF)), which can compromise cap stability [15,19]. MMP release is further amplified by apoptosis of the intimal SMCs and foam cells, contributing to fibrous cap thinning and destabilization [20]. Plaque destabilization leads to exposure of thrombogenic material to the bloodstream, which rapidly triggers platelet aggregation and thrombosis, ultimately compromising arterial lumen size and flow and resulting in ACS [21].

Virmani et al. classified destabilized plaques with thrombi into three distinct histopathologic processes: plaque rupture, plaque erosion and calcific nodules [3]. Plaque rupture refers to a disruption in the fibrous cap that results in continuity between the overlying thrombus and the necrotic core. Lesions with ruptured fibrous cap (RFC) are the most common cause of fatal myocardial infarctions and sudden death [22]. Plaque erosion, or ACS secondary to plaques with intact fibrous cap (IFC), is likely the result of stabilization of plaque, leading to arterial thrombosis without fissuring of the fibrous cap [3]. The key feature of plaque erosion for histopathologic diagnosis is the absence of endothelium, thus exposing the thrombogenic extracellular matrix. The fibrous cap is typically more SMC- and proteoglycan-rich with lower macrophage infiltration [23,24]. A less common cause of coronary thrombosis (in approximately 5% of cases) is due to the protrusion of calcific nodules through a disrupted fibrous cap into the lumen [3,21]. Inflammatory infiltrate is typically absent in this form of plaque destabilization [3].

### 3.1. Plaque Rupture

**Figure 1:**
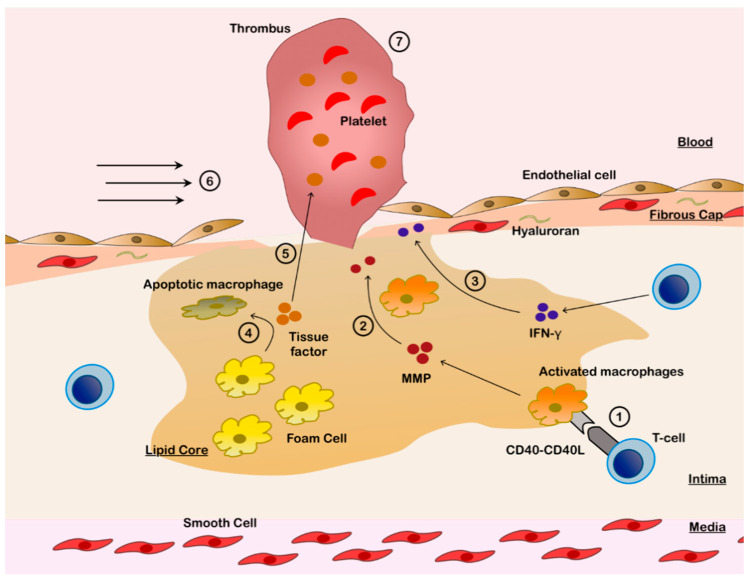
Pathobiology of Plaque Rupture. 1. Infiltrating type 1 T-helper cells activate macrophages in lipid core via CD40L-CD40 interaction. 2. Activated macrophages release matrix metalloproteinases (MMPs), which disrupt the fibrous cap and induce endothelial apoptosis. 3. Infiltrating type 1 T-helper cells release interferon-γ (IFN-γ), which inhibits smooth muscle cells and thereby weakens the fibrous cap. 4. Foam cells become apoptotic due to CD40 stimulation, releasing tissue factor. 5. Tissue factor, which has potent thrombogenic potential, is released into the blood stream. 6. Endothelial apoptosis is augmented by mechanical stress from increased shear forces, especially at the plaque edges, resulting in fibrous cap rupture. 7. Thrombus is formed over the exposed thrombogenic lipidic core.

The underling mechanisms that ultimately lead to rupture in the fibrous cap are summarized in Figure 1. During the evolution of atherogenesis, and as macrophages and T-helper 1 cells continue to infiltrate the fibrous cap, most prominently at the shoulder region of the atheroma [23], “cross-talk” occurs between the innate and adaptive immune systems that results in collagen degradation and weakening of the fibrous cap, leading to rupture in some plaques [25]. T cell-derived CD40 ligands bind to CD40 receptors on macrophages to upregulate the generation of MMPs (specifically collagenases MMP-1, MMP-8 and MMP-13) [26,27]. This process is combined with T-helper 1-mediated release of IFN-γ, which inhibits the function of SMCs to repair and maintain the fibrous cap [25,28] and suppresses the SMC proliferation [20].

Another consequence of CD40 receptor stimulation is tissue factor expression in the apoptotic macrophages (Figure 1), resulting in the generation of tissue factor-enriched microparticles within the lipid core [25]. Plaque rupture exposes the thrombogenic tissue factor to the blood stream, thus activating the extrinsic coagulation pathway and platelet aggregation to initiate thrombus formation. Mechanical stress on plaques is also a significant determinant of plaque destabilization and is observed to be higher in the ruptured cap regions [29]. Higher levels of mechanical stress are concentrated at the edge of the fibrous cap, where the cap is usually the thinnest, compared with the other parts of the cap [30]. Mural shear stress is significantly higher in ACS secondary to RFC as compared to ACS secondary to IFC [31].

### 3.2. Plaque Erosion

In contrast to plaque rupture, plaque erosion is histologically associated with thick cap fibroatheromas and significantly lower degrees of necrosis, hemorrhage or calcification [22,32]. Moreover, plaque erosions are rarely associated with positive remodeling [32] or macrophage infiltration, whilst the underlying plaques have high SMC content in their fibrous cap [23,24,32].

**Figure 2:**
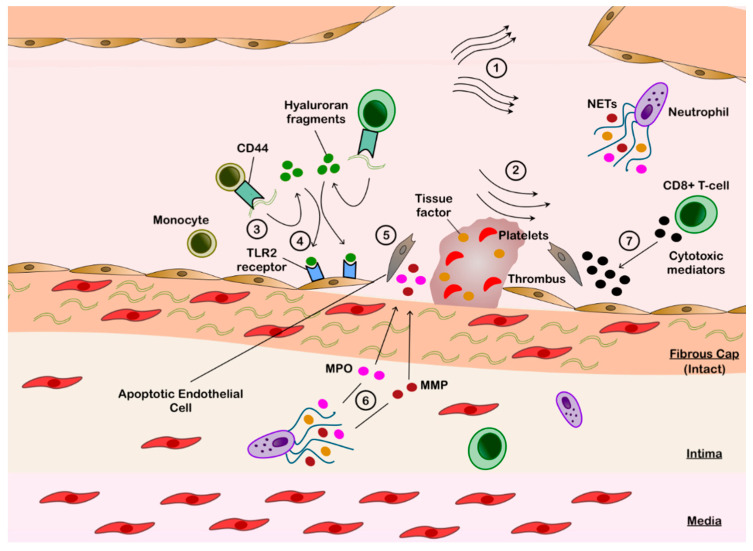
Pathobiology of Plaque Erosion. 1. Impaired laminar flow secondary to bifurcation point. 2. Low shear forces downstream of plaque disrupt cell junctions to induce endothelial apoptosis and mobilize hyaluronan from the fibrous cap. 3. Exposed hyaluronan binds to the circulating monocytes via CD44 and is then hydrolyzed by hyalurorannidase-2 into hyaluronan fragments. 4. Hyaluronan fragments bind to the toll like receptor 2 (TLR2) on endothelial cells. 5. TLR2-stimulated endothelial cells are induced into apoptosis. 6. Endothelial denudation attracts neutrophils, which release neutrophil extracellular traps (NETs) containing metalloproteinase (MMP) and myeloperoxidase (MPO), to augment endothelial apoptosis. Tissue factor is also released in NETs. 7. Infiltrating CD8+ T cells release granzyme A, granulysin and perforin to further disrupt the endothelial layer. 8. Endothelial disruption exposes the underlying fibrous cap, which results in activation of the external coagulation cascade in conjunction with the thrombogenic tissue factor to form thrombus.

The mechanisms for plaque erosion are summarized in Figure 2. Recent evidence suggests that apoptosis of endothelial cells and disruption of endothelial cell-to-extracellular matrix contact are integral in the pathobiology of plaque erosion [33]. There are likely several mechanisms underlying these processes; however, local shear stressors may play a key role in initiating the process [33,34]. Plaque geometry, size and location alter the laminar blood flow and effect the local shear forces [33]. Moreover, intracoronary OCT has highlighted the proximity of the culprit lesions with IFC to coronary branch points, where flow separation is known to cause low sheer stress [35,36]. Low endothelial shear most frequently occurs downstream of plaques and can induce endothelial cell apoptosis [37,38,39]. Increased expression of toll-like receptor 2 (TLR2) has been shown under these conditions [40], which is central in triggering endothelial stress and apoptosis via upregulating MMPs and disrupting endothelial cell junctions by downregulating the vascular endothelial-cadherin complexes [39,41].

TLR2 expression is augmented by neutrophil recruitment, which colocalize to areas of endothelial denudation [35,39]. These exposed regions of sub-endothelial matrix provide a substrate for neutrophil adhesion, activation and degranulation [39]. In vitro studies have shown that TLR2 stimulates neutrophils to further amplify endothelial death through increased expression of MMP [35] and MPO [42]. NETosis is strongly correlated with endothelial cell apoptosis, and it also induces endothelial tissue factor to promote thrombin formation [43,44]. Moreover, NETosis drives platelet activation and aggregation to augment the pro-thrombotic cascade leading to ACS (Figure 2) [45].

Hyaluronan is an abundant proteoglycan macromolecule within the extracellular matrix in the plaques with IFC, in clear distinction with the ruptured plaques where it is almost absent [22]. Given the stark contrast, there has been increasing interest in understanding the role of hyaluronan in the ACS due to IFC. Extracellular hyaluronan exists in its high molecular weight form in homeostasis, but under a local inflammatory environment, it fragments into low molecular weight extracellular molecules that further propagate the inflammatory response [46]. These fragments are involved in neutrophil recruitment and activation [47]. They also serve as endogenous TLR2 ligands to directly induce endothelial cell apoptosis [39,48]. Fragmentation is mediated by peripheral blood monocytes, which have enhanced affinity for high molecular weight hyaluronan via the CD44 receptor in an inflammatory milieu [49] (Figure 2). High molecular weight hyaluronan binds to the cell surface via CD44 and is then hydrolyzed primarily via hyaluroranidase-2 activity to form smaller sized hyaluronan chains before being internalized by monocytes [46]. Hyaluroranidase-2 expression from endothelial cells increases within the low shear environments [50]. Interestingly, expression of hyaluroranidase-2 is higher in OCT-detected ACS secondary to IFC, compared with RFC in the peri-ACS setting, but not at 12-month follow-up [46]. Finally, hyaluronan is functionally prothrombotic, shown to increase platelet adhesion and accelerate fibrin polymerization [51].

More recent findings from the OPTICO-ACS study (Optical Coherence Tomography in Acute Coronary Syndrome) [35], which evaluated culprit lesions of 170 consecutive ACS patients with OCT and simultaneous flow cytometric immunophenotyping, suggest that the adaptive immune system has an integral role in the pathogenesis of IFC-ACS [35]. Local enrichment of CD8+ T cells with higher gradients of cytotoxic effector soluble mediators (granzyme A, granulysin and perforin) has been detected downstream of the culprit lesions with IFC compared to the lesions with RFC [34,35]. Co-culture in vitro studies showed these mediators to display pro-apoptotic effects on endothelial cells. Moreover, the percentage of the adherent CD8+ T cells were significantly increased under in vitro conditions, mimicking the altered flow near bifurcations compared to laminar flow, which was not reflected when monocyte adhesion was investigated. Incubation with anti-integrin β2 or anti-integrin α4 reversed this effect, suggesting that CD8+ T cells display enhanced integrin-dependent endothelial cell adhesion under conditions of disturbed flow [35]. CD44 expression is elevated amongst recruited CD8+ T cells in these conditions [52]. Thus, CD8+ T cells likely work synergistically with peripheral blood monocytes to increase the low molecular weight hyaluronan expression and subsequent TLR2-mediated signaling (Figure 2).

Thus, the available evidence suggests ACS secondary to IFC to be driven by a two-hit mechanism—initial endothelial injury due to low shear forces amplified by TLR2-mediated activity and subsequent neutrophil and CD8+ T cell recruitment to enhance endothelial damage [39]. Exposure of the hyaluronan-rich fibrous cap results in rapidly progressing overlying thrombosis and subsequent ACS.

### 3.3. Calcified Nodules

**Figure 3:**
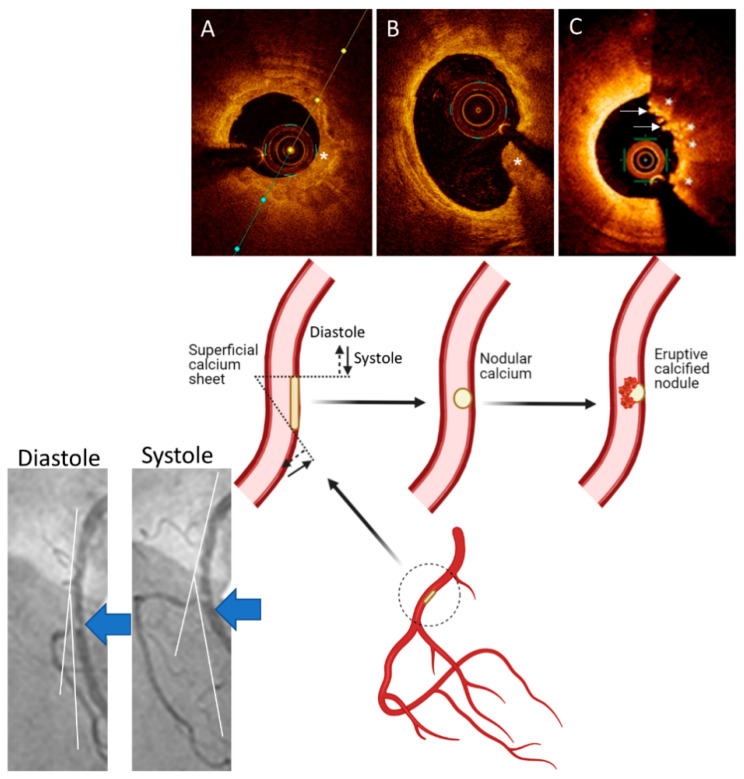
A Working Hypothesis for Formation of Eruptive Calcified Nodules. Superficial calcified sheets (asterisk) (**A**) located in the arterial segments with hinge movement, e.g., in the mid segment of the right coronary artery (large blue arrows on the angiographic pictures and inset), are subject to cyclic mechanical forces during systole and diastole, which could weaken and fragment the sheets of calcium, thus resulting in protruding nodular calcium (asterisk) (**B**) that is surrounded by fibrin. These nodules eventually erupt through the plaque surface ((**C**), asterisks), causing disruption in the intima, with superimposition of thrombus ((**C**), arrows). Parts of the figure are adapted from Lee et al. [55] with permission.

Calcified nodules are the least common atherosclerotic causes of acute coronary thrombosis and are usually found in severely calcified coronary arteries [53]. The mechanisms for formation of the calcified nodules are not known. One working hypothesis is that mechanical stress could fragment sheets of calcium, resulting in small nodules that are surrounded by fibrin that may eventually erupt through the plaque surface [54] (Figure 3). Eruptive calcified nodules typically occur in eccentric lesions where protrusion causes disruption of the overlying endothelium, which is likely to trigger platelet adherence [54]. Eruptive calcified nodules are not to be confused with “nodular calcification” because the latter is not associated with luminal thrombi [3], although it can cause medial wall disruption with rare extension into the adventitia [54]. Calcified nodules are commonly noted in older individuals, most frequently in the mid-right coronary artery (RCA) or left anterior descending (LAD) artery where torsion stress is maximal [3] (Figure 3).

## 4. Clinical and Angiographic Features of ACS According to the Culprit Lesion Subtypes

Early autopsy studies showed ruptured plaque to be the etiology of sudden death in approximately 60–75% of cases where thrombus was present, with plaque erosion and calcified nodules being responsible for almost all the remaining cases [3,24,56]. These studies also recorded higher prevalence of plaque erosion amongst females. A postmortem series on sudden cardiac death revealed the plaque erosions to be almost 3-fold higher compared with ruptured plaque in females (50% vs. 18%, *p* = 0.03) [24].

ST-segment elevation ACS is more prevalent amongst patients with ACS due to lesions with RFC, whilst non-ST-segment elevation ACS are more common in plaques with IFC or calcific plaques [38,57]. More comorbidities, including higher prevalence of hypertension (78.3% vs. 59.3%, *p* < 0.001), diabetes mellitus (40.8% vs. 27.3%, *p* = 0.04) and chronic kidney disease (32.5% vs. 10.9%, *p* < 0.001) are observed in patients with ACS due to plaques with RFC vs. lesions with IFC [38,58,59]. Patients with ACS due to calcific nodules/plaques were the most elderly of the three subgroups and generally present as non-ST-segment elevation ACS [38,59].

On angiographic analysis, patients with RFC-ACS have more complex lesions compared with those with IFC-ACS. Patients with RFC-ACS have higher rates of multivessel disease (39.1% vs. 32.5%, *p* < 0.001) and complex type B2/C lesions (79.6% vs. 60%, *p* < 0.001) [38], with lower survival compared with patients with IFC-ACS at 12 months [60]. The degree of luminal diameter stenosis is significantly greater in the RFC-ACS (83% vs. 77%, *p* < 0.001), and thrombolysis in myocardial infarction (TIMI) flow grading reflects higher rates of absent or faint perfusion distal to the lesions in the RFC-ACS compared with IFC-ACS (TIMI ≤ 1: 39.9% vs. 25.0%, *p* < 0.001) [38].

Culprit lesions are predominantly detected in the proximal and middle LAD artery for both RFC-ACS and IFC-ACS across multiple studies [35,38,57,58]. In patients with RFC-ACS, a higher prevalence of culprit lesions in the RCA is found compared with IFC-ACS (40.5% vs. 28.5%, *p* = 0.002). Lesions with IFC are more prevalent at coronary bifurcations and branch points [58]. A recent study using dual quantitative coronary angiography suggested that lesions with RFC have a larger thrombus volume compared with lesions with IFC (3.48 mm^3^ vs. 1.69 mm^3^, *p* = 0.13) [61]. The prevalence of concentric lesions (symmetrical lesions with only slight irregular borders) is higher in the plaques with IFC, whilst lesions with RFC have a greater proportion of type 2 eccentric lesions (asymmetrical stenosis with irregular borders or scalloped profile) [61,62].

Calcified plaques in ACS present most commonly in the LAD artery and are more likely to involve multiple vessels with greater lesion length compared with both RFC-ACS (multivessel disease: 52% vs. 39%, *p* = 0.004; lesion length: 17.8 mm vs. 16.1 mm, *p* = 0.02) and IFC-ACS (multivessel disease: 52% vs. 32.5%, *p* < 0.001; lesion length: 17.8 mm vs. 15.1 mm, *p* < 0.001) [38].

## 5. Culprit Plaque Evaluation Using Intravascular Imaging

While coronary angiography remains the main imaging modality for assessment of anatomy, it has significant limitations in quantifying functional significance or discerning the etiology of ACS. There is a high interobserver variability in visual estimation of the degree of stenosis [63]. Moreover, on angiography, detection of the non-stenotic atherosclerotic plaques (i.e., plaques that exhibit a high degree of compensatory positive remodeling to maintain the luminal area) is limited [64].

Detailed in vivo characterization of coronary plaque morphology in ACS is feasible by using intravascular imaging modalities, such as IVUS [65] and OCT [57]. Imaging modalities such as near-infrared spectroscopy can identify lipid-rich plaques without defining the morphological structure [66]. During OCT acquisition, near-infrared light spectrum is directed at the vessel wall while the blood is flushed from the coronary artery lumen. OCT generates high-resolution, cross-sectional and three-dimensional images of the vessel. The shorter wavelength of the infrared light used in OCT (1.3 μm) compared with ultrasound waves in IVUS (∼40 μm in soft tissue at 40 MHz) allows for greater axial resolution (10–20 μm vs. 50–150 μm). The axial resolution is improved on high-definition IVUS at 60 MHz (~22 μm) at the expense of reduced depth penetration compared with IVUS at 20 MHz. Overall, OCT light has lower penetration depth than IVUS (1–2 mm vs. 5–6 mm), which limits OCT imaging particularly in the presence of highly attenuating structures, such as red thrombus or lipid and necrotic core [47].

OCT, due to its high axial resolution, is the imaging modality of choice in ACS. On OCT, the inner most tunica intima is visualized as a signal-rich layer, the tunica media as signal-poor, and the outer most adventitia as a signal-rich area [67]. This detailed visualization provides immediate, almost histology grade, definition of the plaque morphology in vivo and aids in elucidating, in real-time, the mechanisms of plaque disruption [67]. OCT has exhibited good inter- and intra-observer reliability in histopathologically controlled studies (κ = 0.83–0.84), with good sensitivity and specificity across multiple plaque morphologies (fibrous, fibrocalcific and lipid-rich plaques) [68,69]. Nevertheless, since image acquisition with OCT in thrombotic culprit lesions usually requires pre-dilatation and/or thrombectomy to restore antegrade flow and improve the quality of OCT images, morphology of the underlying plaque may be distorted. The prevalence of substrates of ACS based on all OCT studies thus far are summarized in Table 1 [33,35,38,57,58,59,70,71,72,73,74,75,76,77,78,79,80,81,82,83,84,85,86]. The total number of lesions with RFC was 3794 (56.9%), the total lesions with IFC was 2222 (33.3%) and the total of calcified nodules was 303 (4.5%), all of which are similar in their overall prevalence to the postmortem histopathologic series.

### 5.1. Plaques with RFC on Intravascular Imaging

**Figure 4:**
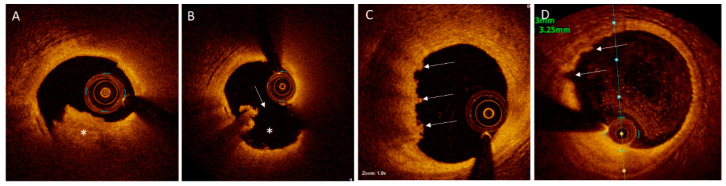
Plaques with Disrupted or Intact Fibrous Cap on Optical Coherence Tomography. (**A**). Mixed white and red thrombus (asterisk) in the left anterior descending (LAD) artery of a patient presenting with ST-segment elevation myocardial infarction. (**B**). After aspiration thrombectomy, a disrupted fibrous cap (arrow) with an empty crater (asterisk), typical for the ruptured plaques, is visualized. (**C**). “Definite” plaque erosion in the LAD artery of a patient presenting with non-ST-segment elevation myocardial infarction. Irregular plaque surface and predominantly white thrombi (arrows) are noted. (**D**). “Probable” plaque erosion is detected in a patient presenting non-ST-segment elevation myocardial infarction by the presence of irregular plaque surface (arrows) without overlying thrombus in the LAD artery.

OCT-defined RFC is characterized by the presence of a thrombus rich in red blood cells (the “red thrombus”) in association with a disrupted fibrous cap that overlies a lipid-rich core with or without cavity formation (Figure 4) [88]. The thrombus appears as a protruding mass within the lumen, causing backscattering of the infrared spectrum emitted by OCT, thus casting a shadow behind the red thrombus. In contrast to a red thrombus, a “white thrombus” is defined as a predominantly platelet-rich thrombus, which generally causes low signal attenuation and is most frequently detected in culprit lesions with IFC (Figure 4).

On OCT, most RFCs are located at the proximal or mid segments of the culprit lesion (80.7% vs. 19.3% in the distal segment) [87]. Compared with RFCs at the proximal or mid segments of culprit lesions, RFCs at the distal segment are associated with higher thrombus volume (4.50 vs. 2.02 mm^3^, *p* = 0.027) and incidence of no re-flow (31.7% vs. 12.8%, *p* = 0.003) [87]. On OCT, frequency of plaques with RFC is not different between diabetics and non-diabetics (74.5% vs. 62.5%, *p* = 0.59) [89], a finding that is in contrast with previous OCT [58] and IVUS-near-infrared spectroscopy-based [90] studies that showed higher frequency of plaque rupture in diabetic patients. With OCT assessment, a clear circadian pattern is recognized in the incidence of plaque rupture, with a peak at 09:00, whereas such pattern is not evident in plaque erosion or calcified nodules [91]. The probability of plaque rupture varies during specific weekdays times, while these patterns do not exist over weekend days. These observations support a role for catecholamine surge in triggering ACS secondary to RFC.

On IVUS, RFC is commonly associated with a vessel wall cavity, formed through downstream embolization of the necrotic core. RFC is detected as an IVUS signal void and with confirmation of the communication between the cavity and the coronary artery by injection of saline or contrast to enhance the visualized contours [92]. Compared with high-resolution imaging with OCT, detection of a thrombus on IVUS is challenging. Assessment of a thrombus by IVUS can be improved by stationary imaging at the site of the suspected thrombus and injection of contrast to better delineate the luminal contour [93]. Moreover, high-definition IVUS provides superior resolution and may improve identification of ruptured plaques, but studies comparing the diagnostic performance of high-definition IVUS with OCT are warranted.

### 5.2. Plaques with IFC on Intravascular Imaging

OCT-defined plaques with IFC are visualized as thrombi overlying an irregular luminal surface, with no evidence of cap rupture on multiple frames (Figure 4) [94]. When the underlying plaque is difficult to assess due to signal attenuation by the thrombus, “probable” IFC is diagnosed if superficial lipid or calcification are absent immediately proximal or distal to the thrombus [93,95]. Probable IFC can also be diagnosed in the setting of an irregular surface in the absence of thrombus (Figure 4). This definition contrasts with early autopsy studies, which mandated the overlying thrombus to be present [57]. The resolution of OCT is not adequate to allow for identification of endothelial cells on plaque surface [96]; therefore, compared with plaque rupture, diagnosis of plaque erosion with OCT remains challenging and needs further validation and refinement. Detection of plaque erosion on IVUS, defined as a lack of “dissection or cleft” at the culprit plaque surface [97], is more challenging than on OCT.

The findings of the recent OPTICO-ACS study (Optical Coherence Tomography in Acute Coronary Syndrome) [35] closely correlated with the histopathologic features, showing that lesions with RFC were more often lipid rich (100% vs. 91%, *p* = 0.01), had greater calcification (mean calcium arc 48 ± 39° vs. 32 ± 33°, *p* = 0.01) and were associated with larger thrombi (thrombus score 126 ± 87 vs. 85 ± 74, *p* = 0.02) compared with the lesions with IFC. Thin cap fibroatheromas were present in almost all lesions with RFC compared to nearly half the lesions with IFC (98% vs. 50%, *p* < 0.01). Lesions with RFC had a lower mean fibrous cap thickness (55.1 ± 7 μm vs. 80.1 ± 55 μm, *p* < 0.001). Mixed thrombus predominated in lesions with RFC (71%), whereas white thrombus was most common in lesions with IFC (69%). Interestingly, there was no red thrombus identified in lesions with IFC; however, this finding may have been confounded by exclusion of *n* = 28 patients because of suboptimal image quality due to marked OCT signal attenuation, likely secondary to red thrombus.

The EROSION study (Effective Anti-Thrombotic Therapy Without Stenting: Intravascular Optical Coherence Tomography-Based Management in Plaque Erosion) [59] evaluated 60 patients presenting with IFC-ACS with OCT at baseline and 1 month after enrollment. Findings in this study also showed white thrombus as the predominant thrombus type (73.3%); however, there was also red thrombus detected in one-fourth of the patients (26.7%). The reason for this large discrepancy is unclear; however, one contributing factor was that the EROSION study was binary in its classification of thrombus and did not report on a mixed phenotype. Macrophage infiltration (signal-rich punctuate regions with heterogenous backward shadowing on OCT [38]) was more frequently detected in RFCs compared with IFCs (79.9% vs. 51.2%, *p* < 0.001 [58] and 80.1% vs. 55.1%, *p* < 0.001 [38]). In patients with ST-segment elevation ACS included in the EROSION study, culprit lesions with RFC had a smaller luminal area compared with the lesions with IFC (1.6 vs. 1.8 mm^2^, *p* = 0.001). Inclusion of patients with non-ST-segment elevation ACS may explain why this finding was not replicated in the OPTICO-ACS study.

### 5.3. Eruptive Calcified Nodules on Intravascular Imaging

At culprit lesion sites, three morphological subtypes of calcium are detected on OCT: eruptive calcified nodules, superficial calcific sheets and calcified protrusions (Figure 3) [38]. Eruptive calcified nodules present as an expulsion of a cluster of small calcified nodules into the lumen by rupturing the fibrous cap (Figure 3), similar to calcified nodules on histopathology [3]. Like lipid-rich lesions with RFC, eruptive calcified nodules are predominantly associated with overlying red thrombus (83.3%) [38]. Imaging of eruptive calcific nodule is possible with IVUS and OCT, with OCT providing superior detection of thrombus, delineation of superficial and deep boundaries of calcium and plaque disruption. However, there are limitations to OCT imaging, for instance the presence of protruding calcium can pose challenges in tissue differentiation, particularly by attenuation of deeper structures, resulting in misrepresentation as red thrombus and potential misdiagnosis of an acute event. Similarly, distinguishing lipid core from calcium if the boundaries are ill-defined or detecting calcium when there is overlying thrombus can be better achieved with IVUS and virtual histology-IVUS [93].

Superficial calcific sheets appear as sheet-like calcific plates without a protruding mass into the lumen and can be associated with RFC or IFC and most commonly are associated with white thrombus (66.3%). Calcified protrusions, referred to as protruding nodular calcifications in histopathology, have a smooth leading edge (Figure 3) and can be associated with IFCs or RFCs. The latter two subtypes of calcific plaques are novel findings, detected with OCT, with no histopathologic studies confirming these subtypes as distinct etiologies of ACS [98]. These calcified plaque subtypes are likely “bystanders” rather than the actual causes of the ACS, but more studies are needed to further elucidate their roles in ACS. Calcific plaques have the least lipidic components and macrophage accumulation compared with the other culprit lesions (RFC-ACS and IFC-ACS). Calcified plaques also have the largest minimal luminal area (1.78 mm^2^ vs. 1.38 mm^2^ vs. 1.43 mm^2^) compared with RFC-ACS (*p* < 0.001) and IFC-ACS (*p* < 0.001), respectively.

## 6. OCT-Guided Treatment of ACS

The ability of OCT to determine the site of plaque rupture or erosion, thrombus burden and the longitudinal extent of underlying plaque, as well as to accurately measure reference lumen and vessel diameters, suggest the utility of OCT in guiding percutaneous coronary intervention (PCI) in ACS. By OCT assessment in TROFI (Randomized study to Assess the Effect of Thrombus Aspiration on Flow Area in Patients With STEMI) in 141 patients with ST-segment elevation ACS, manual thrombectomy did not increase the effective flow area or minimal stent area. [99]. Although routine aspiration thrombectomy is not recommended, a retrospective OCT-based study reported a correlation between post-thrombectomy residual thrombus and the extent of microvascular dysfunction and myocardial damage, suggesting potential use of thrombectomy in lesions with high thrombus burden to reduce distal embolization and preserve microcirculatory function [74] This approach requires prospective validation before adoption in clinical practice. In a retrospective analysis of 588 lesions in 507 patients in CLI-OPCI ACS (Centro per la Lotta Contro L’Infarto-Optimization of Percutaneous Coronary Intervention Database Undergoing PCI for ACS), predictors of stent-related events on OCT were similar to the elective setting: underexpansion (minimal stent area <4.5 mm^2^ (hazard ratio [HR] = 2.72, *p* < 0.01)), stent inflow/outflow disease (reference lumen area <4.5 mm^2^ at the distal (HR = 6.07, *p* < 0.01) or proximal (HR = 8.5, *p* < 0.001) stent edges) and dissection at the distal stent edge >200 μm (HR = 3.84, *p* < 0.001) [100]. Additionally, intrastent plaque or thrombus protrusion (HR = 2.35, *p* < 0.01) was an independent predictor of adverse outcomes [100].

In a 2:1 propensity-matched prospective cohort study, pre- and post-stenting OCT guidance in 214 patients with ST-segment elevation ACS resulted in larger final minimum lumen diameter compared with angiographic guidance in 428 patients (2.99 ± 0.48 mm vs. 2.79 ± 0.47 mm, *p* < 0.0001), potentially because of further post-dilatation in suboptimally deployed stents in the OCT arm [101]. In the randomized DOCTORS study (Does Optical Coherence Tomography Optimize Results of Stenting), higher post-PCI fractional flow reserve values were achieved with OCT guidance vs. angiography guidance in 240 patients with non-ST-segment elevation ACS (0.94 ± 0.04 vs. 0.92 ± 0.05, *p* < 0.005) [102]. In a retrospective analysis of 11,731 patients in the Korea AMI Registry database with acute myocardial infarction who underwent PCI, intravascular modalities were utilized in 2659 (22.7%) patients, including 2333 (19.9%) IVUS and 277 (2.4%) OCT. Compared with a propensity-matched cohort of angiography guidance alone, the patient-oriented composite endpoint (all-cause death, any infarction and any revascularization) was lower with intravascular imaging guidance (5.9% vs. 7.7%; HR = 0.74; 95% confidence interval 0.60–0.92; *p* = 0.006), and the device-oriented composite endpoint (cardiac death, target-vessel reinfarction and target-lesion revascularization; 5.0% vs. 6.8%; HR = 0.72; 95% confidence interval 0.57–0.90; *p* = 0.004) followed a similar trend [103]. Patients with ACS within 24 h of symptom onset were excluded from the large-scale ILUMIEN IV: OPTIMAL PCI trial (Optical Coherence Tomography Guided Coronary Stent Implantation Compared to Angiography: A Multicenter Randomized Trial in PCI) [104] because of the relatively high rates of non-analyzable pre-PCI OCT acquisitions due to high thrombus burden, inefficacy of thrombectomy to reduce the thrombus burden and poor blood clearance [101]. Appropriately powered randomized controlled trials are warranted to substantiate the potential benefits for OCT-guided optimization of PCI in ACS that are suggested by the non-randomized studies.

Immediately post-stenting, unfavorable findings on OCT (e.g., malapposition, tissue protrusion and thrombus burden) were more frequently observed in PCI on plaque rupture compared with plaque erosion [59]. In contrast, strut coverage on OCT in follow-up, with its known caveat as a surrogate for vascular healing around the stent struts [96], was less pronounced in eroded plaques compared with ruptured plaques [105]. Since the fibrous cap is still intact in IFC-ACS, conservative management without exposing patients to the long-term risk of permanent stent placement appears as an appealing strategy if effective reperfusion and favorable long-term outcomes can be achieved. In a cohort of 31 patients, Prati et al. [100] compared a conservative strategy of anti-platelet therapy after thrombectomy without stent placement to the standard practice in ST-segment elevation ACS due to plaques with IFC. Post-procedural residual stenosis and TIMI 3 reperfusion were similar across both groups. After a median follow-up of approximately 2 years, none of the 12 conservatively managed patients required revascularization, while one of the 19 patients from the standard practice cohort required intervention during this follow-up period [100]. It is important to recognize that the study was a prospective non-randomized trial, where the choice of management was at the discretion of the operator, with several potential confounding factors. The conservative group was also on average 10 years younger (52.2 vs. 62.6 years) than the standard therapy group, which may have contributed to the relatively comparable outcomes.

This conservative strategy was subsequently investigated in the single-arm proof-of-concept EROSION study [59] in which patients with OCT-detected IFC-ACS either underwent manual thrombus aspiration or received bolus glycoprotein IIb/IIIa inhibitor. In the instances where TIMI 3 grade reperfusion together with <70% luminal diameter stenosis was achieved, the lesions were not stented, and patients were treated with long-term aspirin and ticagrelor. Of the 60 patients eventually enrolled, 47 met the primary criterion of >50% reduction of thrombus at one-month as assessed by OCT, and 53 patients were free of adverse events (cardiac death, reinfarction, rehospitalization, target lesion revascularization, stroke or major bleeding). In the one-year follow-up study [106], 49 of 53 (92.5%) patients analyzed remained free of events. Among 52 patients who completed a median follow-up of 4.8 years, there were no incidences of death, myocardial infarction, stroke, bypass surgery or heart failure, while 11 (21.1%) patients underwent elective target lesion revascularization [107]. The patients who did not require target lesion revascularization during the follow-up had a larger reduction in thrombus volume on repeat OCT at 1 month compared with the group who required revascularization (95% vs. 45%, *p* = 0.001) [107]. Large randomized controlled trials are warranted to establish whether a no-stenting approach is safe and effective in ACS secondary to plaque erosions.

Given the recent evidence of neutrophil and CD8+ lymphocyte involvement in the pathogenesis of IFC-ACS, targeting inflammatory pathways may be a useful therapeutic approach [35]. The effect of canakinumab (an IL-1β monoclonal antibody) [108] and colchicine [109] in reducing the rates of ischemic cardiovascular events in patients with ACS supports the inflammatory hypothesis in atherothrombosis. The anti-inflammatory effects of colchicine include a reduction in the formation of NETs in patients with ACS undergoing PCI [110]. Long-term therapy with colchicine has resulted in a reduction in the low attenuation plaque volume on CT angiography and in a reduction in ischemic events in patients presenting with ACS [109,111].

Treatment of calcific plaques in ACS remains challenging. Minimal stent area was the smallest in PCI on calcific nodules compared with plaques with IFC or RFC (5.20 mm^2^ vs. 5.44 mm^2^ and 6.44 mm^2^, respectively, *p* = 0.001) [76]. Rates of stent edge dissection were also highest in the OCT-defined calcific nodule compared with the other two lesion subtypes, as were the rates of stent malapposition. The use of rotational or orbital atherectomy may improve these results; however, these devices are contraindicated in thrombotic lesions, while intravascular lithotripsy has not been tested in ACS and may theoretically cause arrhythmias by the R-on-T phenomenon [112]. We have reported deferred intravascular lithotripsy-facilitated stenting after initial establishment of TIMI 3 flow in ST-segment elevation MI due to severely calcified culprit lesion as a safe and effective therapeutic approach [113]; nevertheless, further studies are needed to establish whether this approach or other strategies lead to better procedural and long-term outcomes in this subset of challenging lesions in ACS.

## 7. Conclusions

Mechanistic insights gained by postmortem histopathologic studies have been reproduced by intravascular imaging in vivo, especially by high-resolution OCT imaging. While the culprit lesions in more than half of the patients presenting with ACS are ruptured plaques, plaque erosions are responsible for ACS in approximately one-third of cases. A conservative no-stenting approach with antithrombotic therapy in plaque erosion is supported by small, non-randomized studies and warrants testing in large, randomized trials. Intracoronary imaging with OCT is also a useful adjunctive tool in the diagnosis and appropriate management of the rarer causes of ACS, such as eruptive calcified nodules. Insights gained by the mechanistic studies into the pathogenesis of ACS due to lesions with OCT-defined IFC, in particular the role of innate and adaptive immunity, may provide treatment opportunities beyond coronary intervention and antithrombotic therapies, such as with immune modulation.

## Figures and Tables

**Table 1:** Substrates of Acute Coronary Syndromes on Optical Coherence Tomography.

Study	Cohort	Lesions	RFC-ACS	IFC-ACS	Calcified Nodule	Other Causes
Leistner et al. [33]	ACS	170	98	32	3	37
Guagliumi et al. [70]	STEMI	128	63	32	-	33
Dai et al. [58]	STEMI	822	564	209	5	44
Yamamoto et al. [85]	ACS	1241	607	477	157	-
Jia et al. [57]	ACS	126	55	39	10	22
Chandran et al. [71]	STEMI	40	23	15	-	2
Niccoli et al. [72]	ACS	139	82	57	-	-
Yonetsu et al. [83]	ACS	318	141	131	-	46
Kajander et al. [73]	STEMI	70	34	31	5	-
Kwon et al. [33]	ACS	133	90	43	-	-
Higuma et al. [74]	STEMI	112	72	30	9	1
Kubo et al. [75]	STEMI	30	22	7	-	1
Khalifa et al. [76]	ACS	288	172	82	34	-
Nishiguchi et al. [86]	ACS	326	160	153	-	13
Wang et al. [87]	STEMI	80	37	25	2	16
Fang et al. [79]	STEMI	1442	972	348	23	99
Shibuya et al. [80]	ACS	483	237	218	28	-
Kobayashi et al. [81]	ACS	362	163	149	21	29
Sun et al. [82]	STEMI	211	123	82	6	0
Hu et al. [59]	ACS	141	79	62	-	-
**Total, *n* (%)**		**6662 (100%)**	**3794** **(56.9%)**	**2222** **(33.3%)**	**303** **(4.5%)**	**343** **(5.0%)**

ACS: acute coronary syndromes; IFC-ACS: intact fibrous cap–acute coronary syndrome; RFC-ACS: ruptured fibrous cap–acute coronary syndrome; STEMI: ST-segment elevation myocardial infarction.
